# 5-Chloro-5′′-[4-(di­methyl­amino)­benzyl­idene]-4′-[4-(di­methyl­amino)­phen­yl]-1′,1′′-di­methyl­dispiro­[indoline-3,2′-pyrrolidine-3′,3′′-piperidine]-2,4′′-dione

**DOI:** 10.1107/S1600536813033771

**Published:** 2013-12-18

**Authors:** I. S. Ahmed Farag, Adel S. Girgis, A. A. Ramadan, A. M. Moustafa, Edward R. T. Tiekink

**Affiliations:** a Solid State Department, Physics Division, National Research Centre, Dokki, Giza, Egypt; b Pesticide Chemistry Department, National Research Centre, Dokki, Giza 12622, Egypt; c Physics Department, Faculty of Science, Helwan University, Helwan, Cairo, Egypt; d Department of Chemistry, University of Malaya, 50603 Kuala Lumpur, Malaysia

## Abstract

The title compound, C_34_H_38_ClN_5_O_2_, has spiro links connecting the pyrrolidine ring and indole residue, as well as the piperidine and pyrrolidine rings. A half-chair conformation is found for the piperidine ring with the C atom connected to the spiro-C atom lying 0.738 (4) Å out of the plane of the remaining five atoms (r.m.s. deviation = 0.0407 Å). The methyl­ene C atom is the flap in the envelope conformation for the pyrrolidine ring. In the crystal, supra­molecular chains are sustained by alternating eight-membered {⋯HNCO}_2_ and 14-membered {⋯HC_5_O}_2_ synthons. Chains are connected into a three-dimensional network by (pyrrolidine-bound phenyl-meth­yl)C—H⋯π(pyrrolidine-bound phen­yl) edge-to-face inter­actions.

## Related literature   

For the biological activity of related spiro pyrrolidine analogues, see: Girgis *et al.* (2012 ▶); Kumar *et al.* (2008 ▶). For related structural studies, see: Ahmed Farag *et al.* (2013*a* ▶,*b* ▶). For the synthesis of the precursor mol­ecule, see: Al-Omary *et al.* (2012 ▶).
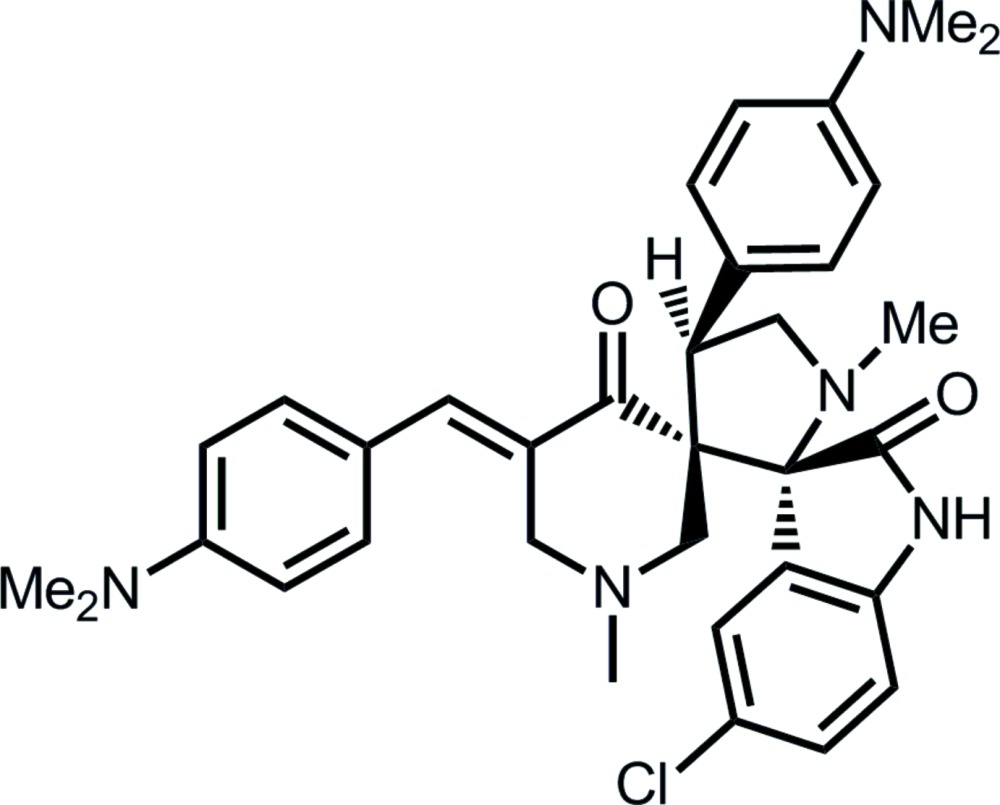


## Experimental   

### 

#### Crystal data  

C_34_H_38_ClN_5_O_2_
*M* *_r_* = 584.14
Triclinic, 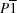

*a* = 11.5458 (5) Å
*b* = 12.2357 (5) Å
*c* = 12.5267 (7) Å
α = 64.341 (2)°
β = 84.286 (2)°
γ = 83.467 (2)°
*V* = 1582.29 (13) Å^3^
*Z* = 2
Mo *K*α radiation
μ = 0.16 mm^−1^
*T* = 293 K
0.31 × 0.18 × 0.13 mm

#### Data collection  

Enraf–Nonius 590 KappaCCD diffractometer
Absorption correction: multi-scan (*SADABS*; Sheldrick, 1996 ▶) *T*_min_ = 0.782, *T*_max_ = 0.927
13814 measured reflections
7127 independent reflections
2244 reflections with *I* > 2σ(*I*)
*R*_int_ = 0.081

#### Refinement  

*R*[*F* ^2^ > 2σ(*F* ^2^)] = 0.061
*wR*(*F* ^2^) = 0.165
*S* = 0.91
7127 reflections
385 parameters
H-atom parameters constrained
Δρ_max_ = 0.15 e Å^−3^
Δρ_min_ = −0.20 e Å^−3^

### 

Data collection: *COLLECT* (Hooft, 1998 ▶); cell refinement: *DENZO* (Otwinowski & Minor, 1997 ▶) and *COLLECT*; data reduction: *DENZO* and *COLLECT*; program(s) used to solve structure: *SHELXS97* (Sheldrick, 2008 ▶); program(s) used to refine structure: *SHELXL97* (Sheldrick, 2008 ▶); molecular graphics: *ORTEP-3 for Windows* (Farrugia, 2012 ▶), *DIAMOND* (Brandenburg, 2006 ▶) and *Qmol* (Gans & Shalloway, 2001 ▶); software used to prepare material for publication: *publCIF* (Westrip, 2010 ▶).

## Supplementary Material

Crystal structure: contains datablock(s) general, I. DOI: 10.1107/S1600536813033771/hg5368sup1.cif

Structure factors: contains datablock(s) I. DOI: 10.1107/S1600536813033771/hg5368Isup2.hkl

Additional supporting information:  crystallographic information; 3D view; checkCIF report

## Figures and Tables

**Table 1:** Hydrogen-bond geometry (Å, °) *Cg*1 is the centroid of the C27–C32 ring.

*D*—H⋯*A*	*D*—H	H⋯*A*	*D*⋯*A*	*D*—H⋯*A*
N4—H4*n*⋯O2^i^	0.86	2.00	2.853 (4)	169
C28—H28⋯O1^ii^	0.93	2.47	3.337 (4)	156
C33—H33c⋯*Cg*1^iii^	0.96	2.88	3.807 (5)	163

Symmetry codes: (i) 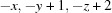; (ii) 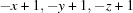; (iii) 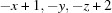.

**Fig. 1.**
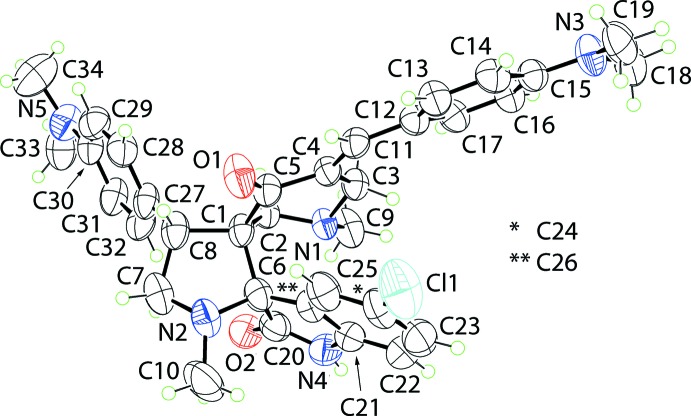
The molecular structure of (I) showing the atom-labelling scheme and displacement ellipsoids at the 50% probability level.

**Fig. 2.**
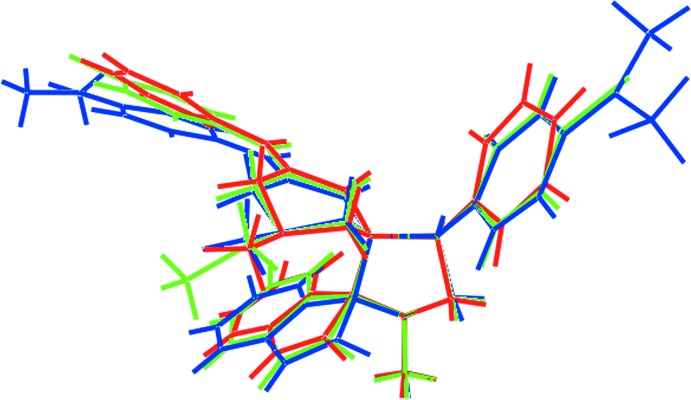
Overlay diagram of the three recently determined compounds, drawn so that the central pyrrolidine rings are overlapped. Red image (Ahmed Farag et al., 2013a), green image (Ahmed Farag et al., 2013b) and blue image (present study).

**Fig. 3.**
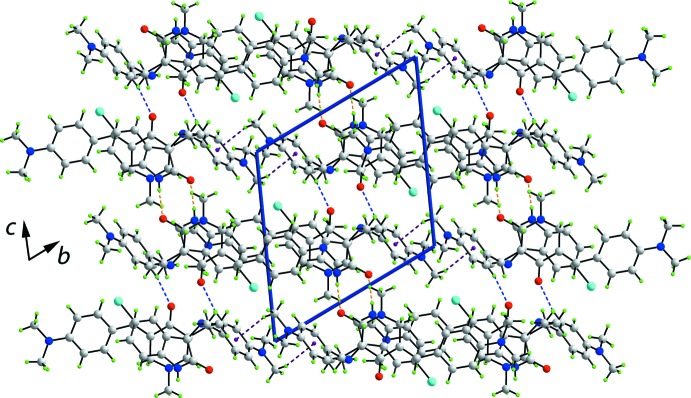
A view of the unit-cell contents in projection down the a axis in (I). The N—H···O and π—π interactions are shown as orange and purple dashed lines, respectively.

## supplementary crystallographic information

### 2.1. Synthesis and crystallization

A mixture of equimolar amounts of 3*E*,5*E*-1-methyl-3,5-bis­(4-di­methyl­amino­phenyl­methyl­idene)-4-piperidones (5 mmol), prepared by a literature procedure (Al-Omary *et al.*, 2012), 5-chloro­isatin and sarcosine in absolute ethanol (25 ml) was boiled under reflux (TLC monitoring). The separated solid was collected and crystallized from *n*-butanol affording (I). Reaction time 20 h. Yellow crystals. *M*.pt: 525–527 K. Yield 68%. Anal. Calcd. for C_34_H_38_ClN_5_O_2_ (584.17): C, 69.91; H, 6.56; N, 11.99. Found: C, 70.02; H, 6.68; N, 11.93. IR: ν_max_/cm^-1^: 3168 (N—H); 1692 (C═O); 1613, 1566 (C═C).

### 2.2. Refinement

The C-bound H atoms were geometrically placed (C—H = 0.93–0.98 Å) and refined as riding with *U_iso_*(H) = 1.2–1.5*U_eq_*(C). The N-bound H-atom was treated similarly with N—H = 0.86 Å, and with *U_iso_*(H) = 1.2*U_eq_*(N).

### 3. Results and discussion

In continuation of our biological and crystallographic studies of spiro­pyrrolidine derivatives derivatives (Girgis *et al.* 2012; Ahmed Farag *et al.* 2013*a*), which are known to to have biological activity (Kumar *et al.* 2008), the title compound, (I), was synthesised and characterised crystallographically.

The molecular structure of (I) is shown in Fig. 1 which shows two spiro links, *i.e*. at atom C1, linking the piperidine and pyrrolidine rings, and at atom C6 where the pyrrolidine ring and indole residue are connected. The piperidine ring carries phenyl­methyl­idene and pyrrolidine-bound aryl residues at positions C4 and C8, respectively. An *E* conformation is found the C4═C11 double bond. The piperidine-N1 atom has *sp*^3^ character as seen by the sum of the angles at this atom of 337 °. A half-chair conformation is found for the piperidine ring in which the C2 atom lies 0.738 (4) Å out of the plane of the remaining five atoms (r.m.s. deviation = 0.0407 Å). With respect to the piperidine ring, both the N-bound methyl and phenyl­methyl­idene substituents occupy equatorial positions . An envelope conformation is found for the pyrrolidine ring with the C7 being the flap atom lying 0.547 (5) Å out of the plane of the remaining four atoms which have a r.m.s. deviation of 0.0906 Å. The similarity of the molecular structures of (I) and recently described derivatives (Ahmed Farag *et al.* 2013*a*,*b*), at least in terms of the cores of these, is emphasised in the overlay diagram, Fig. 2.

The crystal structure of (I) features centrosymmetric eight-membered {···HNCO}_2_ synthons, Table 1. These are linked into supra­molecular chains aligned in the (1 1 2) plane by 14-membered {···HC_5_O}_2_ synthons, Table 1. Chains are connected into the three-dimensional architecture by (pyrrolidine-bound phenyl-methyl)C–H···π(pyrrolidine-bound phenyl), edge-to-face, inter­actions, Fig. 3 and Table 1.

### Crystal data

**Table:**

C_34_H_38_ClN_5_O_2_	*Z* = 2
*M**_r_* = 584.14	*F*(000) = 620
Triclinic, *P*1	*D*_x_ = 1.226 Mg m^−^^3^
Hall symbol: -P 1	Mo *K*α radiation, λ = 0.71073 Å
*a* = 11.5458 (5) Å	Cell parameters from 5831 reflections
*b* = 12.2357 (5) Å	θ = 2.9–27.5°
*c* = 12.5267 (7) Å	µ = 0.16 mm^−^^1^
α = 64.341 (2)°	*T* = 293 K
β = 84.286 (2)°	Block, orange
γ = 83.467 (2)°	0.31 × 0.18 × 0.13 mm
*V* = 1582.29 (13) Å^3^	

### Data collection

**Table:**

Enraf–Nonius 590 KappaCCD diffractometer	7127 independent reflections
Radiation source: fine-focus sealed tube	2244 reflections with *I* > 2σ(*I*)
Graphite monochromator	*R*_int_ = 0.081
φ and ω scans	θ_max_ = 27.5°, θ_min_ = 3.1°
Absorption correction: multi-scan (*SADABS*; Sheldrick, 1996)	*h* = −14→14
*T*_min_ = 0.782, *T*_max_ = 0.927	*k* = −15→15
13814 measured reflections	*l* = −16→10

### Refinement

**Table:**

Refinement on *F*^2^	Primary atom site location: structure-invariant direct methods
Least-squares matrix: full	Secondary atom site location: difference Fourier map
*R*[*F*^2^ > 2σ(*F*^2^)] = 0.061	Hydrogen site location: inferred from neighbouring sites
*wR*(*F*^2^) = 0.165	H-atom parameters constrained
*S* = 0.91	*w* = 1/[σ^2^(*F*_o_^2^) + (0.055*P*)^2^] where *P* = (*F*_o_^2^ + 2*F*_c_^2^)/3
7127 reflections	(Δ/σ)_max_ < 0.001
385 parameters	Δρ_max_ = 0.15 e Å^−^^3^
0 restraints	Δρ_min_ = −0.20 e Å^−^^3^

### Special details

**Table:**

Geometry. All s.u.'s (except the s.u. in the dihedral angle between two l.s. planes) are estimated using the full covariance matrix. The cell s.u.'s are taken into account individually in the estimation of s.u.'s in distances, angles and torsion angles; correlations between s.u.'s in cell parameters are only used when they are defined by crystal symmetry. An approximate (isotropic) treatment of cell s.u.'s is used for estimating s.u.'s involving l.s. planes.
Refinement. Refinement of *F*^2^ against ALL reflections. The weighted *R*-factor *wR* and goodness of fit *S* are based on *F*^2^, conventional *R*-factors *R* are based on *F*, with *F* set to zero for negative *F*^2^. The threshold expression of *F*^2^ > 2σ(*F*^2^) is used only for calculating *R*-factors(gt) *etc*. and is not relevant to the choice of reflections for refinement. *R*-factors based on *F*^2^ are statistically about twice as large as those based on *F*, and *R*- factors based on ALL data will be even larger.

### Fractional atomic coordinates and isotropic or equivalent isotropic displacement parameters (Å^2^)

**Table:**

	*x*	*y*	*z*	*U*_iso_*/*U*_eq_	
Cl1	0.00842 (11)	0.90513 (9)	0.33316 (9)	0.1119 (5)	
O1	0.3446 (2)	0.59561 (19)	0.5160 (2)	0.0750 (8)	
O2	0.1064 (2)	0.4073 (2)	0.9448 (2)	0.0704 (7)	
N1	0.2654 (2)	0.6453 (2)	0.8157 (2)	0.0469 (7)	
N2	0.1139 (3)	0.4251 (3)	0.6910 (2)	0.0662 (9)	
N3	0.3573 (3)	1.3398 (2)	0.3237 (2)	0.0652 (8)	
N4	0.0072 (3)	0.5930 (3)	0.8421 (3)	0.0668 (9)	
H4n	−0.0348	0.5979	0.9008	0.080*	
N5	0.6847 (3)	0.0837 (3)	1.0145 (3)	0.0800 (10)	
C1	0.2785 (3)	0.5106 (3)	0.7211 (3)	0.0504 (9)	
C2	0.3223 (3)	0.5320 (3)	0.8207 (2)	0.0482 (9)	
H2A	0.3038	0.4658	0.8968	0.058*	
H2B	0.4063	0.5362	0.8108	0.058*	
C3	0.3109 (3)	0.7505 (3)	0.7153 (2)	0.0494 (9)	
H3A	0.3852	0.7653	0.7346	0.059*	
H3B	0.2575	0.8215	0.7032	0.059*	
C4	0.3277 (3)	0.7353 (3)	0.6021 (3)	0.0452 (8)	
C5	0.3213 (3)	0.6136 (3)	0.6040 (3)	0.0507 (9)	
C6	0.1383 (3)	0.5203 (3)	0.7259 (3)	0.0560 (10)	
C7	0.1985 (3)	0.3228 (3)	0.7457 (3)	0.0749 (12)	
H7A	0.2022	0.2669	0.7090	0.090*	
H7B	0.1794	0.2792	0.8300	0.090*	
C8	0.3149 (3)	0.3828 (3)	0.7230 (3)	0.0613 (10)	
H8	0.3435	0.3973	0.6420	0.074*	
C9	0.2653 (3)	0.6588 (3)	0.9254 (3)	0.0725 (11)	
H9A	0.2272	0.5934	0.9884	0.109*	
H9B	0.2244	0.7350	0.9160	0.109*	
H9C	0.3443	0.6570	0.9443	0.109*	
C10	−0.0077 (4)	0.3941 (3)	0.7112 (4)	0.1041 (15)	
H10A	−0.0166	0.3365	0.6800	0.156*	
H10B	−0.0579	0.4663	0.6721	0.156*	
H10C	−0.0283	0.3590	0.7948	0.156*	
C11	0.3454 (2)	0.8291 (3)	0.4941 (3)	0.0490 (9)	
H11	0.3555	0.8061	0.4318	0.059*	
C12	0.3515 (3)	0.9578 (3)	0.4575 (3)	0.0474 (9)	
C13	0.3543 (3)	1.0342 (3)	0.3361 (3)	0.0551 (9)	
H13	0.3540	0.9997	0.2830	0.066*	
C14	0.3575 (3)	1.1582 (3)	0.2919 (3)	0.0561 (10)	
H14	0.3596	1.2047	0.2102	0.067*	
C15	0.3576 (3)	1.2159 (3)	0.3670 (3)	0.0500 (9)	
C16	0.3594 (3)	1.1397 (3)	0.4884 (3)	0.0530 (9)	
H16	0.3623	1.1737	0.5415	0.064*	
C17	0.3571 (3)	1.0161 (3)	0.5311 (3)	0.0543 (10)	
H17	0.3593	0.9690	0.6125	0.065*	
C18	0.3406 (3)	1.3962 (3)	0.4056 (3)	0.0846 (13)	
H18A	0.2661	1.3786	0.4480	0.127*	
H18B	0.3435	1.4826	0.3621	0.127*	
H18C	0.4013	1.3646	0.4607	0.127*	
C19	0.3349 (3)	1.4166 (3)	0.2012 (3)	0.0779 (12)	
H19A	0.3928	1.3961	0.1509	0.117*	
H19B	0.3382	1.5002	0.1859	0.117*	
H19C	0.2588	1.4047	0.1852	0.117*	
C20	0.0856 (3)	0.4990 (4)	0.8513 (3)	0.0599 (10)	
C21	0.0032 (3)	0.6814 (3)	0.7249 (3)	0.0568 (10)	
C22	−0.0627 (3)	0.7898 (4)	0.6808 (4)	0.0724 (11)	
H22	−0.1086	0.8163	0.7316	0.087*	
C23	−0.0601 (3)	0.8595 (4)	0.5598 (4)	0.0775 (12)	
H23	−0.1035	0.9342	0.5283	0.093*	
C24	0.0067 (4)	0.8180 (3)	0.4863 (3)	0.0696 (11)	
C25	0.0755 (3)	0.7089 (3)	0.5291 (3)	0.0667 (11)	
H25	0.1204	0.6821	0.4780	0.080*	
C26	0.0748 (3)	0.6413 (3)	0.6511 (3)	0.0545 (9)	
C27	0.4107 (4)	0.3056 (3)	0.8031 (3)	0.0573 (10)	
C28	0.5228 (4)	0.2936 (3)	0.7547 (3)	0.0693 (11)	
H28	0.5377	0.3369	0.6735	0.083*	
C29	0.6123 (4)	0.2206 (3)	0.8222 (4)	0.0689 (11)	
H29	0.6851	0.2146	0.7852	0.083*	
C30	0.5965 (4)	0.1550 (3)	0.9452 (4)	0.0645 (11)	
C31	0.4844 (4)	0.1671 (3)	0.9943 (3)	0.0681 (11)	
H31	0.4694	0.1245	1.0756	0.082*	
C32	0.3959 (3)	0.2404 (3)	0.9255 (3)	0.0669 (11)	
H32	0.3231	0.2467	0.9623	0.080*	
C33	0.6631 (4)	0.0129 (3)	1.1413 (4)	0.0925 (13)	
H33A	0.6249	0.0649	1.1757	0.139*	
H33B	0.7360	−0.0223	1.1773	0.139*	
H33C	0.6142	−0.0506	1.1545	0.139*	
C34	0.7943 (4)	0.0575 (4)	0.9629 (4)	0.1278 (18)	
H34A	0.7817	0.0180	0.9139	0.192*	
H34B	0.8444	0.0049	1.0247	0.192*	
H34C	0.8301	0.1318	0.9155	0.192*	

### Atomic displacement parameters (Å^2^)

**Table:**

	*U*^11^	*U*^22^	*U*^33^	*U*^12^	*U*^13^	*U*^23^
Cl1	0.1734 (13)	0.0715 (7)	0.0785 (8)	−0.0019 (7)	−0.0476 (8)	−0.0139 (6)
O1	0.128 (2)	0.0533 (14)	0.0436 (15)	−0.0158 (14)	0.0206 (15)	−0.0242 (12)
O2	0.0789 (19)	0.0616 (16)	0.0541 (16)	−0.0088 (14)	0.0129 (14)	−0.0119 (13)
N1	0.067 (2)	0.0403 (16)	0.0340 (16)	−0.0073 (14)	0.0040 (14)	−0.0171 (13)
N2	0.084 (2)	0.0508 (19)	0.070 (2)	−0.0043 (19)	−0.0158 (18)	−0.0298 (16)
N3	0.098 (2)	0.0430 (18)	0.0476 (19)	−0.0053 (16)	−0.0090 (17)	−0.0116 (16)
N4	0.065 (2)	0.065 (2)	0.063 (2)	−0.0093 (18)	0.0162 (17)	−0.0227 (18)
N5	0.086 (3)	0.078 (2)	0.075 (3)	0.005 (2)	−0.002 (2)	−0.035 (2)
C1	0.070 (3)	0.041 (2)	0.039 (2)	−0.0017 (18)	0.0080 (19)	−0.0191 (16)
C2	0.062 (2)	0.041 (2)	0.038 (2)	−0.0047 (17)	0.0013 (17)	−0.0141 (16)
C3	0.061 (2)	0.0426 (19)	0.041 (2)	−0.0002 (17)	−0.0038 (17)	−0.0146 (16)
C4	0.054 (2)	0.045 (2)	0.039 (2)	−0.0054 (17)	0.0056 (17)	−0.0213 (17)
C5	0.064 (2)	0.046 (2)	0.040 (2)	−0.0014 (18)	0.0042 (19)	−0.0183 (18)
C6	0.071 (3)	0.050 (2)	0.052 (2)	−0.010 (2)	0.000 (2)	−0.0258 (19)
C7	0.108 (3)	0.054 (2)	0.071 (3)	−0.011 (3)	−0.009 (2)	−0.032 (2)
C8	0.089 (3)	0.051 (2)	0.045 (2)	0.001 (2)	0.006 (2)	−0.0245 (18)
C9	0.118 (3)	0.058 (2)	0.042 (2)	−0.017 (2)	0.008 (2)	−0.0220 (19)
C10	0.102 (4)	0.086 (3)	0.140 (4)	−0.029 (3)	−0.031 (3)	−0.053 (3)
C11	0.055 (2)	0.049 (2)	0.041 (2)	−0.0022 (18)	0.0066 (17)	−0.0208 (17)
C12	0.053 (2)	0.045 (2)	0.041 (2)	−0.0061 (17)	0.0014 (17)	−0.0155 (18)
C13	0.071 (3)	0.054 (2)	0.042 (2)	−0.0079 (19)	0.0039 (18)	−0.0218 (18)
C14	0.068 (3)	0.055 (2)	0.035 (2)	−0.0096 (19)	0.0049 (18)	−0.0099 (18)
C15	0.056 (2)	0.046 (2)	0.045 (2)	−0.0046 (18)	−0.0027 (18)	−0.0161 (19)
C16	0.067 (3)	0.048 (2)	0.042 (2)	−0.0052 (19)	−0.0084 (18)	−0.0173 (18)
C17	0.066 (3)	0.049 (2)	0.042 (2)	−0.0083 (19)	−0.0037 (18)	−0.0116 (18)
C18	0.123 (4)	0.053 (2)	0.076 (3)	−0.010 (2)	0.002 (3)	−0.027 (2)
C19	0.100 (3)	0.045 (2)	0.069 (3)	−0.010 (2)	−0.015 (2)	−0.003 (2)
C20	0.063 (3)	0.058 (3)	0.059 (3)	−0.015 (2)	0.006 (2)	−0.025 (2)
C21	0.052 (3)	0.053 (2)	0.065 (3)	−0.007 (2)	−0.002 (2)	−0.023 (2)
C22	0.060 (3)	0.070 (3)	0.088 (3)	0.000 (2)	0.000 (2)	−0.037 (3)
C23	0.070 (3)	0.065 (3)	0.097 (4)	0.001 (2)	−0.024 (3)	−0.031 (3)
C24	0.087 (3)	0.050 (3)	0.068 (3)	−0.009 (2)	−0.022 (3)	−0.017 (2)
C25	0.085 (3)	0.060 (3)	0.060 (3)	−0.010 (2)	−0.015 (2)	−0.026 (2)
C26	0.063 (3)	0.047 (2)	0.055 (3)	−0.0063 (19)	−0.006 (2)	−0.021 (2)
C27	0.083 (3)	0.041 (2)	0.041 (2)	0.005 (2)	0.007 (2)	−0.0154 (18)
C28	0.096 (3)	0.051 (2)	0.052 (3)	−0.011 (2)	0.020 (3)	−0.017 (2)
C29	0.075 (3)	0.066 (3)	0.064 (3)	−0.005 (2)	0.014 (2)	−0.030 (2)
C30	0.081 (3)	0.047 (2)	0.066 (3)	0.006 (2)	−0.007 (3)	−0.026 (2)
C31	0.094 (3)	0.052 (2)	0.048 (3)	0.010 (2)	0.004 (3)	−0.017 (2)
C32	0.087 (3)	0.058 (2)	0.047 (3)	0.002 (2)	0.019 (2)	−0.021 (2)
C33	0.122 (4)	0.071 (3)	0.085 (3)	0.016 (3)	−0.029 (3)	−0.035 (3)
C34	0.095 (4)	0.143 (5)	0.135 (5)	0.033 (3)	−0.008 (4)	−0.060 (4)

### Geometric parameters (Å, º)

**Table:**

Cl1—C24	1.744 (4)	C11—H11	0.9300
O1—C5	1.214 (3)	C12—C13	1.398 (4)
O2—C20	1.244 (4)	C12—C17	1.397 (4)
N1—C2	1.444 (4)	C13—C14	1.375 (4)
N1—C9	1.453 (4)	C13—H13	0.9300
N1—C3	1.461 (3)	C14—C15	1.400 (4)
N2—C7	1.450 (4)	C14—H14	0.9300
N2—C10	1.465 (4)	C15—C16	1.399 (4)
N2—C6	1.472 (4)	C16—C17	1.371 (4)
N3—C15	1.370 (4)	C16—H16	0.9300
N3—C19	1.442 (4)	C17—H17	0.9300
N3—C18	1.452 (4)	C18—H18A	0.9600
N4—C20	1.350 (4)	C18—H18B	0.9600
N4—C21	1.398 (4)	C18—H18C	0.9600
N4—H4n	0.8600	C19—H19A	0.9600
N5—C30	1.368 (4)	C19—H19B	0.9600
N5—C34	1.430 (5)	C19—H19C	0.9600
N5—C33	1.453 (4)	C21—C22	1.365 (5)
C1—C2	1.523 (4)	C21—C26	1.388 (4)
C1—C5	1.542 (4)	C22—C23	1.379 (5)
C1—C8	1.563 (4)	C22—H22	0.9300
C1—C6	1.606 (4)	C23—C24	1.369 (5)
C2—H2A	0.9700	C23—H23	0.9300
C2—H2B	0.9700	C24—C25	1.387 (5)
C3—C4	1.499 (4)	C25—C26	1.387 (4)
C3—H3A	0.9700	C25—H25	0.9300
C3—H3B	0.9700	C27—C32	1.392 (4)
C4—C11	1.358 (4)	C27—C28	1.393 (4)
C4—C5	1.490 (4)	C28—C29	1.375 (5)
C6—C26	1.515 (4)	C28—H28	0.9300
C6—C20	1.549 (4)	C29—C30	1.399 (5)
C7—C8	1.547 (4)	C29—H29	0.9300
C7—H7A	0.9700	C30—C31	1.397 (5)
C7—H7B	0.9700	C31—C32	1.375 (4)
C8—C27	1.512 (4)	C31—H31	0.9300
C8—H8	0.9800	C32—H32	0.9300
C9—H9A	0.9600	C33—H33A	0.9600
C9—H9B	0.9600	C33—H33B	0.9600
C9—H9C	0.9600	C33—H33C	0.9600
C10—H10A	0.9600	C34—H34A	0.9600
C10—H10B	0.9600	C34—H34B	0.9600
C10—H10C	0.9600	C34—H34C	0.9600
C11—C12	1.446 (4)		
			
C2—N1—C9	114.3 (3)	C12—C13—H13	118.6
C2—N1—C3	112.1 (2)	C13—C14—C15	121.5 (3)
C9—N1—C3	110.9 (2)	C13—C14—H14	119.2
C7—N2—C10	114.6 (3)	C15—C14—H14	119.2
C7—N2—C6	107.0 (3)	N3—C15—C16	122.1 (3)
C10—N2—C6	115.4 (3)	N3—C15—C14	121.8 (3)
C15—N3—C19	120.4 (3)	C16—C15—C14	116.1 (3)
C15—N3—C18	119.4 (3)	C17—C16—C15	121.7 (3)
C19—N3—C18	117.1 (3)	C17—C16—H16	119.2
C20—N4—C21	111.3 (3)	C15—C16—H16	119.2
C20—N4—H4n	124.4	C16—C17—C12	122.8 (3)
C21—N4—H4n	124.4	C16—C17—H17	118.6
C30—N5—C34	121.1 (4)	C12—C17—H17	118.6
C30—N5—C33	120.8 (4)	N3—C18—H18A	109.5
C34—N5—C33	116.9 (4)	N3—C18—H18B	109.5
C2—C1—C5	106.3 (2)	H18A—C18—H18B	109.5
C2—C1—C8	115.7 (3)	N3—C18—H18C	109.5
C5—C1—C8	111.4 (3)	H18A—C18—H18C	109.5
C2—C1—C6	111.1 (3)	H18B—C18—H18C	109.5
C5—C1—C6	108.0 (3)	N3—C19—H19A	109.5
C8—C1—C6	104.1 (2)	N3—C19—H19B	109.5
N1—C2—C1	107.8 (3)	H19A—C19—H19B	109.5
N1—C2—H2A	110.2	N3—C19—H19C	109.5
C1—C2—H2A	110.2	H19A—C19—H19C	109.5
N1—C2—H2B	110.2	H19B—C19—H19C	109.5
C1—C2—H2B	110.2	O2—C20—N4	125.1 (3)
H2A—C2—H2B	108.5	O2—C20—C6	125.9 (4)
N1—C3—C4	113.6 (2)	N4—C20—C6	108.9 (3)
N1—C3—H3A	108.9	C22—C21—C26	121.5 (4)
C4—C3—H3A	108.9	C22—C21—N4	128.7 (4)
N1—C3—H3B	108.9	C26—C21—N4	109.7 (3)
C4—C3—H3B	108.9	C21—C22—C23	119.1 (4)
H3A—C3—H3B	107.7	C21—C22—H22	120.4
C11—C4—C5	116.6 (3)	C23—C22—H22	120.4
C11—C4—C3	123.2 (3)	C24—C23—C22	119.6 (4)
C5—C4—C3	120.1 (2)	C24—C23—H23	120.2
O1—C5—C4	121.9 (3)	C22—C23—H23	120.2
O1—C5—C1	120.2 (3)	C23—C24—C25	122.3 (4)
C4—C5—C1	117.8 (3)	C23—C24—Cl1	119.6 (4)
N2—C6—C26	111.2 (3)	C25—C24—Cl1	118.1 (4)
N2—C6—C20	112.8 (3)	C26—C25—C24	117.6 (4)
C26—C6—C20	100.7 (3)	C26—C25—H25	121.2
N2—C6—C1	102.7 (3)	C24—C25—H25	121.2
C26—C6—C1	118.5 (3)	C25—C26—C21	119.8 (4)
C20—C6—C1	111.4 (3)	C25—C26—C6	131.0 (4)
N2—C7—C8	103.5 (3)	C21—C26—C6	109.1 (3)
N2—C7—H7A	111.1	C32—C27—C28	115.3 (4)
C8—C7—H7A	111.1	C32—C27—C8	124.8 (4)
N2—C7—H7B	111.1	C28—C27—C8	120.0 (3)
C8—C7—H7B	111.1	C29—C28—C27	122.7 (3)
H7A—C7—H7B	109.0	C29—C28—H28	118.6
C27—C8—C7	115.2 (3)	C27—C28—H28	118.6
C27—C8—C1	117.2 (3)	C28—C29—C30	121.6 (4)
C7—C8—C1	104.4 (3)	C28—C29—H29	119.2
C27—C8—H8	106.4	C30—C29—H29	119.2
C7—C8—H8	106.4	N5—C30—C31	121.3 (4)
C1—C8—H8	106.4	N5—C30—C29	122.7 (4)
N1—C9—H9A	109.5	C31—C30—C29	116.0 (4)
N1—C9—H9B	109.5	C32—C31—C30	121.6 (4)
H9A—C9—H9B	109.5	C32—C31—H31	119.2
N1—C9—H9C	109.5	C30—C31—H31	119.2
H9A—C9—H9C	109.5	C31—C32—C27	122.8 (4)
H9B—C9—H9C	109.5	C31—C32—H32	118.6
N2—C10—H10A	109.5	C27—C32—H32	118.6
N2—C10—H10B	109.5	N5—C33—H33A	109.5
H10A—C10—H10B	109.5	N5—C33—H33B	109.5
N2—C10—H10C	109.5	H33A—C33—H33B	109.5
H10A—C10—H10C	109.5	N5—C33—H33C	109.5
H10B—C10—H10C	109.5	H33A—C33—H33C	109.5
C4—C11—C12	132.2 (3)	H33B—C33—H33C	109.5
C4—C11—H11	113.9	N5—C34—H34A	109.5
C12—C11—H11	113.9	N5—C34—H34B	109.5
C13—C12—C17	115.1 (3)	H34A—C34—H34B	109.5
C13—C12—C11	118.0 (3)	N5—C34—H34C	109.5
C17—C12—C11	126.9 (3)	H34A—C34—H34C	109.5
C14—C13—C12	122.7 (3)	H34B—C34—H34C	109.5
C14—C13—H13	118.6		

### Hydrogen-bond geometry (Å, º)

Cg1 is the centroid of the C27–C32 ring.

**Table:**

*D*—H···*A*	*D*—H	H···*A*	*D*···*A*	*D*—H···*A*
N4—H4*n*···O2^i^	0.86	2.00	2.853 (4)	169
C28—H28···O1^ii^	0.93	2.47	3.337 (4)	156
C33—H33c···*Cg*1^iii^	0.96	2.88	3.807 (5)	163

Symmetry codes: (i) −*x*, −*y*+1, −*z*+2; (ii) −*x*+1, −*y*+1, −*z*+1; (iii) −*x*+1, −*y*, −*z*+2.
